# Stingray Sensor System for Persistent Survey of the GEO Belt

**DOI:** 10.3390/s24082596

**Published:** 2024-04-18

**Authors:** Tanner Campbell, Adam Battle, Dan Gray, Om Chabra, Scott Tucker, Vishnu Reddy, Roberto Furfaro

**Affiliations:** 1 Department of Aerospace & Mechanical Engineering, The University of Arizona, Tucson, AZ 85721, USA; 2 Lunar & Planetary Laboratory, The University of Arizona, Tucson, AZ 85721, USA; adambattle@arizona.edu (A.B.); vishnureddy@arizona.edu (V.R.); 3 Sidereal Technology, Estacada, OR 97023, USA; grayarea56@gmail.com; 4 Department of Computer Science, The University of Illinois Urbana-Champaign, Urbana, IL 61801, USA; ochabra@arizona.edu; 5 Starizona, Tucson, AZ 85704, USA; scott@starizona.com; 6 Department of Systems & Industrial Engineering, The University of Arizona, Tucson, AZ 85721, USA

**Keywords:** SSA, astrometry, photometry, survey, array, wide field, GEO belt

## Abstract

The Stingray sensor system is a 15-camera optical array dedicated to the nightly astrometric and photometric survey of the geosynchronous Earth orbit (GEO) belt visible above Tucson, Arizona. The primary scientific goal is to characterize GEO and near-GEO satellites based on their observable properties. This system is completely autonomous in both data acquisition and processing, with human oversight reserved for data quality assurance and system maintenance. The 15 ZWO ASI1600MM Pro cameras are mated to Sigma 135 mm f/1.8 lenses and are controlled simultaneously by four separate computers. Each camera is fixed in position and observes a 7.6-by-5.8-degree portion of the GEO belt, for a total of a 114-by-5.8-degree field of regard. The GAIA DR2 star catalog is used for image astrometric plate solution and photometric calibration to GAIA G magnitudes. There are approximately 200 near-GEO satellites on any given night that fall within the Stingray field of regard, and all those with a GAIA G magnitude brighter than approximately 15.5 are measured by the automated data reduction pipeline. Results from an initial one-month survey show an aggregate photometric uncertainty of 0.062 ± 0.008 magnitudes and astrometric accuracy consistent with theoretical sub-pixel centroid limits. Provided in this work is a discussion of the design and function of the system, along with verification of the initial survey results.

## 1. Introduction

Space situational awareness (SSA) is the collection and maintenance of knowledge of objects in space near the Earth. Most resident space objects (RSOs) near the Earth are either too small or too far away to be reliably resolved by all but the most advanced ground-based sensors. As such, the characterization of these objects must rely on “unresolved” techniques to learn as much information as possible from the point-source RSO. In particular, satellites in or near to the geosynchronous Earth orbit (GEO) are critical to SSA due to the prolific exploitation of that region of space [1].

The physical characterization of RSOs ultimately enables their unique identification and can give critical insight into their behavior [2,3,4]. Characterization techniques often include photometric and spectroscopic studies that aim to understand the behavior of light as it interacts with the object in question [5,6,7,8]. Traditionally, photometric surveys of the GEO belt visible from a given location on the ground have used two modes of operation: (1) performing a “hopscotch” through the RSOs each night when using a single telescope, only collecting a few observations of each RSO before moving on to the next; or (2) staying fixed on one or a few RSOs in the telescope field of view each night for the whole night. Skipping between RSOs all night allows for a shorter “revisit time”, obtaining at least some data on most, if not all, desired RSOs each night. However, the data duration per target is short, which makes characterization and behavior/change analysis challenging, since these quite often employ data-heavy approaches [9,10]. Moreover, any amount of time spent not observing a particular RSO represents gaps in knowledge, which adversely impact characterization. On the other hand, observing one or a few RSOs all night long allows for a longer duration of data collection per target, making characterization more informative. This is not without drawbacks, though, as the longer observing time per target means that fewer RSOs can be observed, and the “revisit time” for subsequent observations of the same RSO could be on the order of a year if one is trying to observe all visible GEO satellites looking at only one per night. Obviously, then, the ideal scenario is to be able to observe all the desired RSOs simultaneously all the time, so that there are no knowledge gaps, and a sufficient quantity of data is gathered for any desired analysis.

Having real-time data on each GEO satellite all night long, every night, would be the most ideal scenario for many SSA applications. All-night data on GEO satellites have been leveraged to show unique identification [11,12] and to learn details of the payload and solar panel operation life cycle of the satellite [13,14]. Maneuvers can also be estimated from these data [15,16]. More intricate analyses have been able to recover shape, mass, material properties, conglomerate abstract concepts like behavior, and more [3,17,18,19,20,21,22]. These data can also serve as a proving ground for new methods of observation association and orbit determination [23,24].

The Stingray system was motivated by Dr. Dave Monet, who had experience while at the U.S. Naval Observatory using a multi-aperture system to observe GEO objects (private communication). The goal for Stingray is to use the data produced to assist in the validation of both physics-based and machine learning-based algorithms and to fuel SSA research for the future. In survey collection mode, Stingray collects data on roughly 200 satellites every night. The metrics collected are mainly position, time, and brightness (astrometry and photometry), which are the required inputs to many of the aforementioned characterization techniques. With persistent coverage of such a diverse set of targets, the Stingray system is ideal for supporting this SSA research. In this work, we present the design and deployment of the Stingray system, as well as initial survey results.

The remainder of this paper is organized as follows: the Stingray system design and architecture is given in Section 2, with an overview of hardware trade studies and prototype systems, as well as a discussion on the data collection and processing methodologies. In Section 3, an analysis of the results from the commissioning and initial survey phases of deployment are presented along with a sample case of data collected on a tumbling GEO satellite. Finally, a summary of the system and results are given in Section 4.

## 2. Design Methodology and System Description

### 2.1. Goals and Objectives

The Stingray sensor system was designed to give persistent observational custody of RSOs in orbit around the Earth near the geosynchronous regime without being prohibitively expensive (i.e., using commercial off-the-shelf equipment) and to address the issues with observation cadence outlined in Section 1. Forefront in the design was also the desire for completely autonomous data collection and processing. With the large number of targets and persistent nighttime operation requirements, human-in-the-loop at any stage of operation would not be feasible. With these design criteria met, the desire is for a system capable of producing reliable astrometric and photometric observations of all the near-GEO RSOs visible to the system above Tucson, AZ.

The goal is to then use this accumulation of metric observations of near-GEO RSOs to drive future cutting-edge research on space object characterization. Moreover, there are many current techniques proposed in the literature, particularly machine learning (ML), that have shown great results using simulations to produce the data required. But, in the case of ML, where often very large data sets are required, often no such real data set exists. These techniques can benefit greatly with access to real data for validation to further improve their applications. Using the data from the Stingray system, we hope to validate and improve many physics- and ML-based characterization techniques in the future.

Being capable of producing near-real-time astrometry and photometry of all satellites in near-geosynchronous orbit simultaneously, the Stingray system creates a huge wealth of data and prompt results. The multi-aperture design gives the best of both modes of survey operation; not only can Stingray maintain all-night custody of the target near-GEO objects, but it can observe them simultaneously without having to repoint or “hopscotch” around the sky. Having access to not just nightly but real-time observations of every near-GEO satellite above a location on the ground opens the door for new types of periodic characterization analyses that focus on instantaneous, short-term, and long-term effects for GEO objects en masse and allows for the analysis or alerting of behavior in an operational cadence that has not previously been accessible to researchers.

Machine learning has been shown to be a very effective tool for satellite characterization [4,17,18,19,21,22], but one of the biggest limiting factors in the development of these algorithms for real-world use is the availability of training data of both sufficient quality and volume. Historically, this has been very difficult to accomplish due to the volume of data required for traditional ML algorithms and the comparatively low throughput of traditional telescope systems. The large amount of data produced by Stingray, however, enables training of ML models for SSA with real data. One of the primary goals of the Stingray system is to address this shortcoming and provide these much-needed data. The data from Stingray also provide an avenue for the validation of physics-based models simulating the translational, rotational, or reflective dynamics of RSOs [21,22,25].

### 2.2. System Design

Since one of the primary motivating factors in the Stingray sensor design was the mitigation of the shortcomings of a traditional telescope system when trying to conduct a survey of the GEO belt (as discussed in Section 1), the obvious first step is to consider multiple apertures/telescopes. A naive solution, then, is to assign one telescope per object of interest. In this way, persistent custody is guaranteed, but this obviously scales poorly as the cost and logistical complexity grow quite rapidly. A contrasting approach would be to try and cover the desired field of regard (FOR) (approximately 120 degrees) with as few sensors as possible. This approach is not without issue, however, as very large field of view sensors are prone to image distortion effects, poor astrometric resolution, detector size becomes a concern, and data processing becomes far more difficult.

For the Stingray system, we chose the latter approach and conducted trade studies of different configurations to optimize the sensor system architecture given our problem criteria. In the following sections, a summary of the trade studies conducted as well as details of prototype systems are presented.

#### 2.2.1. Hardware Trade Study

Two cameras were considered during the design of the Stingray system: the QHY600 (Sony IMX455 CMOS) and the ZWO ASI1600 MM Pro (Panasonic MN34230 CMOS). The QHY600 has a 9576 × 6388 full-frame sensor with 3.76 μm pixels. The ZWO ASI1600MM Pro has a 4656 × 3520 full-frame sensor with 3.8 μm pixels. At the time of consideration, the QHY detector cost just over four times as much as the ZWO. It would also produce approximately 3.75 times the data volume per camera compared to the ZWO. Moreover, Starizona is a local vendor for ZWO cameras, so any warranty or service could be taken care of locally, without the need to ship any cameras. After consideration of the specifications of each sensor, cost, availability, data volume, support, and the desired ultimate design, the ZWO 1600 MM Pro camera was chosen over the QHY600.

A trade study of multiple commercial off-the-shelf (COTS) camera lenses was conducted to evaluate on-sky performance, cost, FOV, and image quality, along with several other metrics important to the ultimate survey data quality. Based on the FOV of the chosen lenses, the system could then be constructed to physically observe all of the FOR. The initial goal was to be able to observe all of the GEO belt above Tucson, AZ, which is more than 30 degrees above the local horizon. This means that the target FOR for the system was 120 degrees wide on the sky. The vertical span of the FOR was of a lower priority because the majority of the target RSOs are geostationary and those that are not (geosynchronous) are observable during their ascending and descending node crossings. Details of the lenses considered during the trade study are given in Table 1.

**Figure 1:**
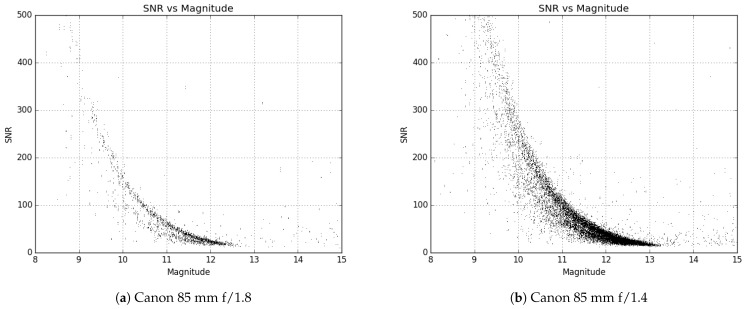

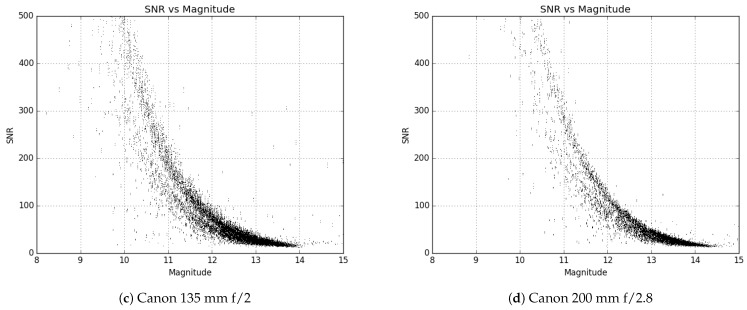
Limiting magnitude plots for some of the lenses tested during the Stingray trade study.

**Figure 2:**
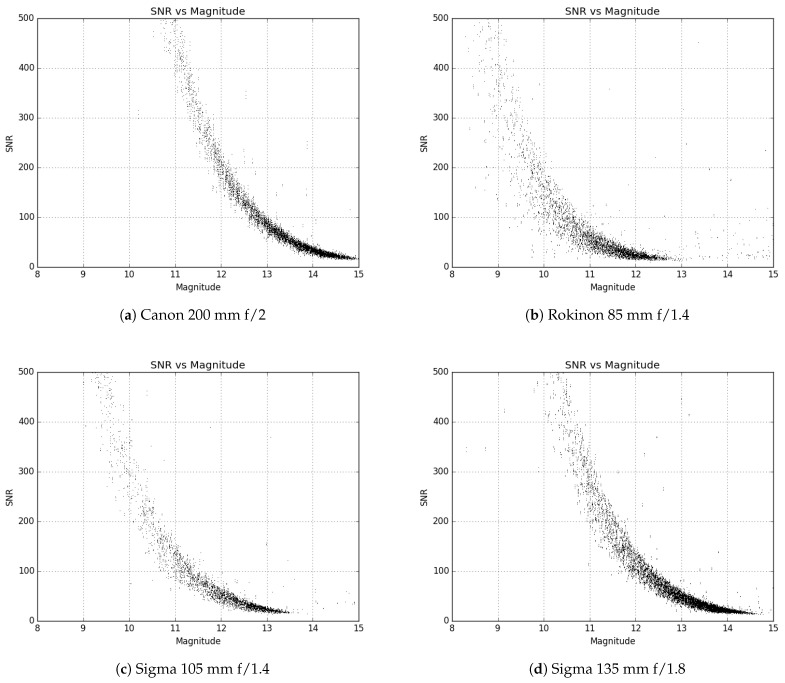
Limiting magnitude plots for some of the lenses tested during the Stingray trade study.

During the trade study of the various lenses, several samples were ordered to evaluate on-sky performance. After initial testing and down selection, multiple examples of candidate lenses were ordered to test consistency. Evaluation of the limiting magnitude of each lens was of paramount importance, as this is what determines the faintest satellites that can be seen in a given image. Many GEO satellites can be relatively faint (>13 V mag), and the aperture of these lenses is very small compared to traditional telescopes, so this test was critical to ensure the desired data could be collected reliably. However, while the aperture would control which satellites could be detected, the field of view (FOV) given by each lens would dictate how many systems were required to cover the desired patch of sky (last column in Table 1). Too many systems would not only increase the cost significantly but would make the operational logistics very complicated as well. In Figure 1 and Figure 2, the limiting magnitude is shown for each lens tested as a plot of the signal-to-noise ratio (SNR) of a source versus its magnitude.

Figure 1 and Figure 2 are created by attaching each of the lenses one at a time to the ZWO ASI1600MM Pro camera and then taking images of the sky field of a standard star. For this, the Landolt standard star fields SA92, SA93, SA96, SA97, SA99, and SA115 were used [26]. Using the data processing process outlined in Section 2.3.3, the images were plate solved, and all stars identified in the images that were matched to a catalog source were measured. The measured SNR was then plotted against the published catalog magnitude for each star resulting in Figure 1 and Figure 2.

**Figure 3:**
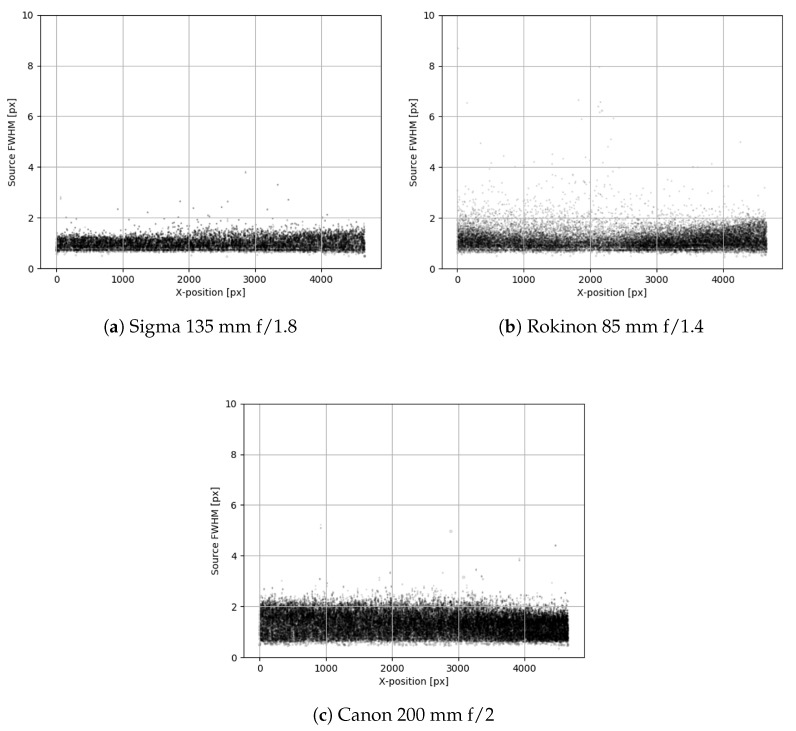
These figures show one way in which the lenses were evaluated for field distortion and optical quality. The FWHM of all sources in a star field was plotted against the source’s x-axis position in the frame. Each marker is scaled in size proportional to the ellipticity of the source in the image. More consistent FWHM and marker size means better results, and any bias present is undesirable. Only three lenses are shown here as an example.

Ultimately after several sample lenses and on-sky tests, the Sigma 135 mm f/1.8 lens was chosen. This lens gave very little edge distortion, had excellent clarity, and the relatively large aperture yielded a competitive limiting magnitude. We would like to note that both the Sigma lenses (105 mm and 135 mm) have the same aperture (75 mm); however, the limiting magnitude of the Sigma 135 mm lens is 14.5, which is a whole magnitude fainter than the Sigma 105 mm. Looking at Table 1, it is obvious that the focal length and pixel scale (which is dependent on focal length) plays an important role in the limiting magnitude of a system. In Figure 3, a comparison of the full width at half the maximum value (FWHM) against the location in the image is given for sources in star field images taken with three lenses. The Sigma lens performs very well in this test, having a very consistent FWHM of all sources and no appreciable spatial biases in the distribution of source metrics. In addition, the 7.6 × 5.8 degree FOV meant that 16 systems would be required to cover the desired FOR. This is much more manageable and cost effective than the 24 required by the other lenses (Canon f/2 and f/2.8) with a similar limiting magnitude.

#### 2.2.2. Stingray Prototype

Based on the results of the trade study, several single-camera prototype systems were made with ZWO ASI1600 MM Pro cameras and Sigma 135 mm f/1.8 lenses. One system was to be affixed to the Leo-20 telescope (IAU MPC code V17) to test the system’s performance on a variety of targets. The second system was mounted to a tripod so that it was mobile but could be used to evaluate static-staring system performance and aid in algorithm development. Others were used to experiment with simultaneous multiple-camera control since no commercially available software was capable of connecting to and controlling more than two of these cameras simultaneously from the same machine.

**Figure 4:**
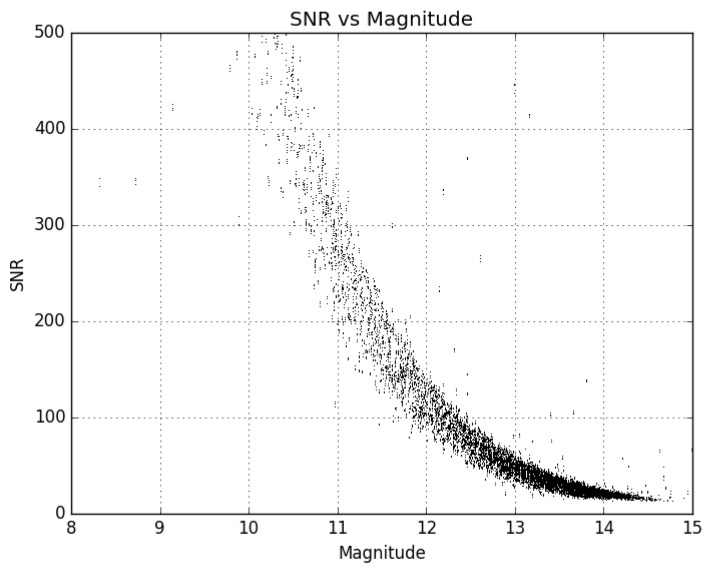
This shows the limiting magnitude for the Stingray system using the Sigma 135 mm f/1.8 lens on a ZWO ASI1600MM Pro Camera during the trade study.

The limiting magnitude of the Stingray prototype system at the V17 telescope site is reproduced from Figure 2 here in Figure 4. During testing, this prototype system was used to observe several types of Earth-orbiting satellites. Some of the results of these experiments observing Starlink low Earth orbit (LEO) satellites are published in [27].

### 2.3. System Architecture

Once decisions could be made based on the results of the trade studies and prototype systems, the full system could be assembled. After hardware selection, there was still work to be performed concerning the automated operation and analysis. During this “engineering” phase of deployment, Stingray was operated every night while software development and hardware tuning took place. Altogether, over 200 nights of testing were performed while fine-tuning the system. In the sections that follow, the chosen hardware architecture is described, as is an overview of the system operation and data processing software that was developed.

#### 2.3.1. Hardware Selection

**Figure 5:**
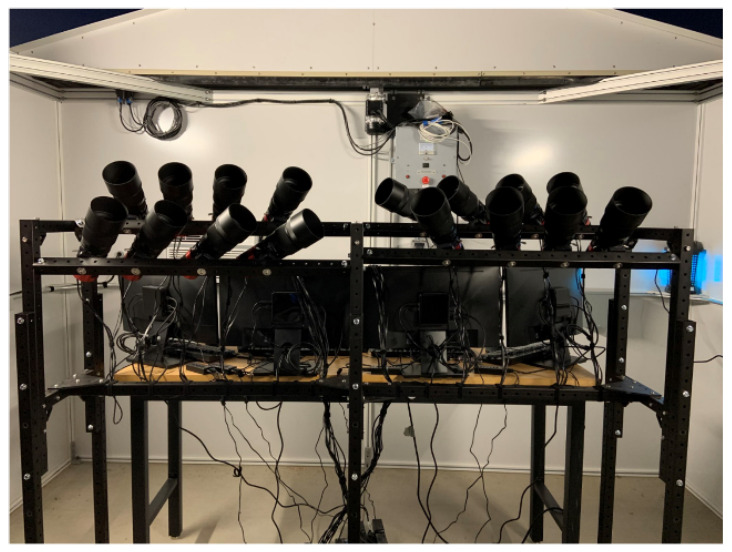
Assembled Stingray system at the Biosphere 2 facility.

The final Stingray system is comprised of 15 ZWO ASI1600MM Pro cameras, each mated to a Sigma 135 mm f/1.8 lens. Shown in Figure 5 is the Stingray system as it was first implemented, with 16 cameras. After the initial mockup, it was decided to raise one of the rows of cameras (back right in Figure 5) to observe lower in the eastern part of the sky (<20 deg elevation). As shown, these cameras were observing low in the western sky over the Pacific Ocean, where there are few geostationary satellites. By raising these cameras and pointing them to the east, they could observe more objects than they were previously able to. However, due to mechanical constraints, one camera had to be removed.

All 15 cameras are mechanically fixed in position, each staring at a different section of the GEO belt visible above Tucson, AZ. Using 600 nm as an approximate central wavelength (halfway between the detector peak quantum efficiency and the GAIA G central wavelength), the 75 mm aperture of the Sigma 135 mm lens gives a theoretical resolving power of 2 arcseconds, which is roughly equivalent to the final system’s local atmospheric seeing. However, the detector pixel pitch is 3.8 μm, giving a pixel scale of 5.9 arcseconds per pixel ax 1× binning, which ultimately constrains the astrometric resolution in this case.

**Figure 6:**
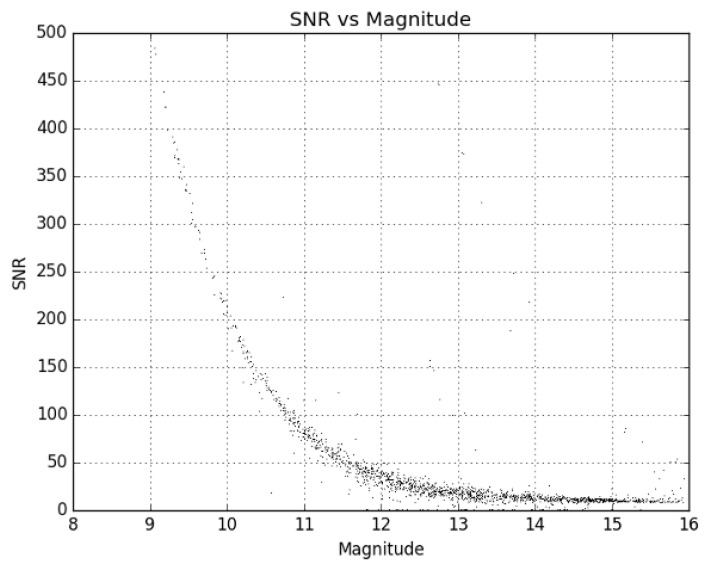
This shows the limiting magnitude for the Stingray system using Sigma 135 mm f/1.8 lenses on ZWO ASI1600MM Pro Cameras from the Biosphere 2 facility. Note that the limiting magnitude is approximately 1 magnitude fainter than it was at the testing site (V17).

The limiting magnitude of the system was found to be just brighter than 16 GAIA G magnitude (Figure 6) from the Biosphere 2 site, which is approximately 1 magnitude fainter than it was found to be during testing. The Biosphere 2 site is a darker site than the V17 observatory, which was used during hardware testing, explaining the gain in limiting magnitude. There is a summary of relevant system details given in Table 2.

#### 2.3.2. Data Collection

To control all fifteen cameras simultaneously, four separate Intel NUC computers are used, each connected to four cameras (one connected to only three), and a fifth Intel NUC is used to act as a central node. Since the entire system is automated, the central node is responsible for monitoring the weather in real time, opening and closing the roof when appropriate, and issuing start/stop/pause image collection commands to the four camera control nodes. Each of the camera control nodes, upon receipt of a command from the central node, performs the appropriate action with each of its cameras. Image plate solution and photometric calibration can happen either in real time on each camera control computer or in batch at the end of the night. As a precaution, real-time updates on weather, roof status, image sequence state, and data processing as well as an end-of-the-night summary of data collected along with visual proof of roof closure (photo) are all sent from the central node using Slack for easy remote system monitoring and alerts.

**Figure 7:**
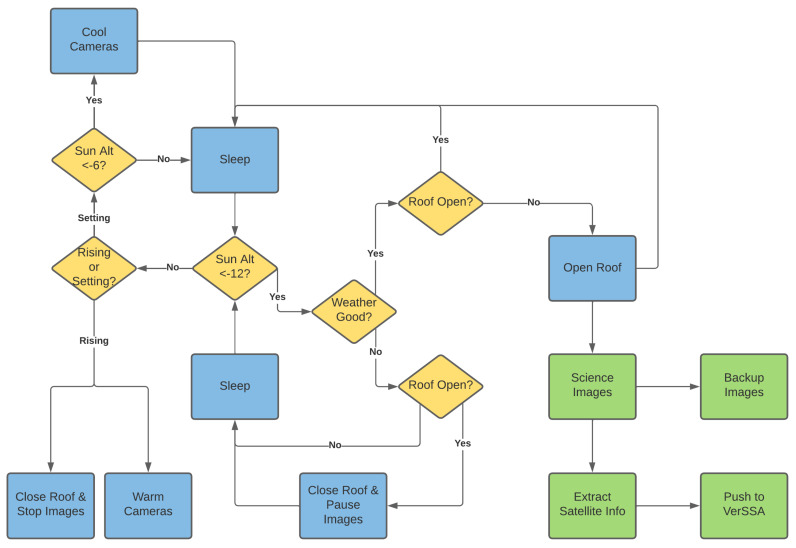
Flow of the control logic for the Stingray system central node. The start of the logic is at the ‘Sun Alt <−12?’ decision block.

Since Stingray is a completely autonomous system, by default, data are to be collected every night. The central node that handles the weather monitoring makes the decision on when/if to open the roof for data acquisition on a given night. Nominally, image collection is set to begin every night when the Sun is 12 deg below the local horizon. However, if there are clouds overhead or other adverse weather conditions (i.e., wind, rain, high humidity, etc.), the central node will delay the start until the conditions clear. Similarly, if the conditions worsen at any point during the night, the central node can pause the image collection and close the roof if necessary to wait for the conditions to improve. Any time the central node makes the decision to close the roof for adverse weather conditions, image collection is paused, but it can be restarted if the roof opens again during the night. In this way, some data can be collected even on less-than-optimal nights, while maintaining system safety and without having to have an operator manually monitor the system and environment all night, every night. A summary of the control logic of the Stingray central node is given in Figure 7. Yellow diamonds represent decision points in the control node logic, blue blocks represent abstract functions that are executed post-decision, and green blocks are processes that take place off of the central control node, such as science image collection, which is executed by the camera control nodes.

During the commissioning and testing phase, Stingray collected over 500,000 images. To help limit the volume of data produced during initial testing, the image collection cadence was a pair of images every 5 min using 2 × 2 binning. A pair of images (one 20 s and one 2 s exposure) was chosen to help in the quality analysis of the system during the commissioning phase. The 2 s exposure images have sufficiently round point spread functions (PSFs) from the stars in-frame to act as a set of “control” images that can be used to validate data processing and to check for any issues, such as focus drift, poor calibration, etc.

Nominally, the observation plan for the GEO survey is a single image (10 s exposure) every minute from all cameras, with 2 × 2 binning. For the CMOS-based ZWO cameras chosen, binning above 2 × 2 is performed in the software and does not provide the same SNR advantages found in typical CCD systems. Coupled with the SNR increase from 2 × 2 binning, which will improve photometric measurements, there is a fourfold reduction in data volume. This reduction significantly eases the burden of data management, cutting the average nightly production to approximately 50 GB with the aforementioned collection cadence. One possible concern from binning the data is the increase in pixel scale (11.8 as/px vs. 5.9 as/px). Since each pixel is larger, astrometric precision is reduced. However, as shown in Section 3.1, the astrometric measurements are still acceptable.

#### 2.3.3. Data Processing and Handling

**Figure 8:**
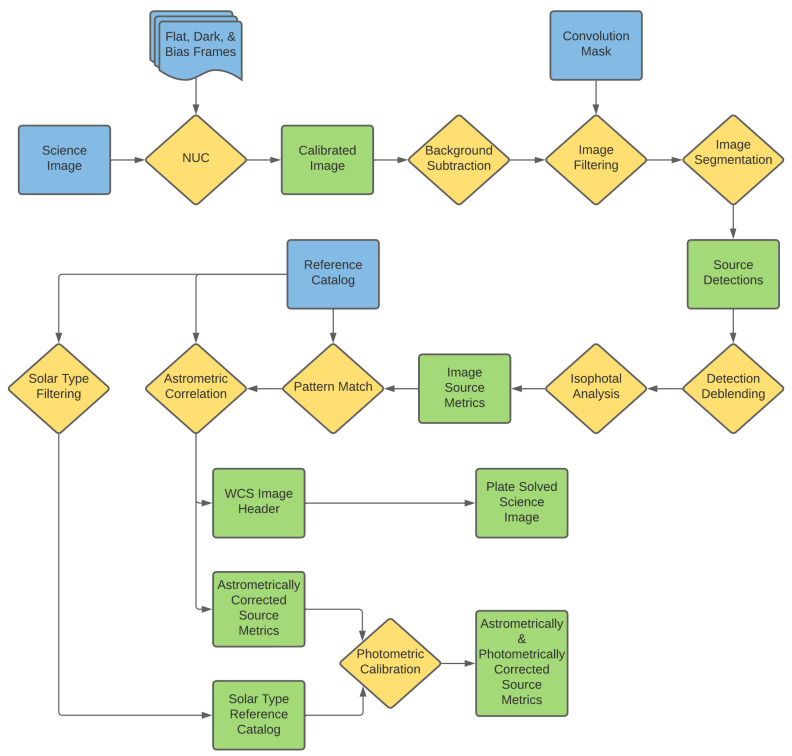
Flow of the astrometric and photometric data processing logic for a single image from the Stingray system.

The Stingray system can process data in two modes. The image nonuniformity corrections (NUCs), plate solutions, and photometric calibrations can all be performed independently, either in real time as images are collected or in batches at any later point. The desired data products from this survey are satellite metrics (angular position and brightness), and these can be extracted from the images also either in real time or in batches; though, due to the stochastic nature of the automated satellite extraction process, it is often more robust to perform the processing in at least small batches as opposed to on an image-by-image basis. The processing can be thought of as being split into three phases: nonuniformity correction, astrometric reduction, and photometric calibration. Details of each phase follow and are presented in order of their application. A diagram of the data reduction process (excluding satellite extraction) starting with a single science image is shown in Figure 8. This is shown for a single image as an example, as some, or all, of the process can be completed in parallel on multiple images simultaneously in a variety of ways. Yellow diamonds represent an abstract function/process that is completed during the data analysis, blue blocks represent a data product that is used as an external input to the processing pipeline, and green blocks represent a data product that is either internal to or produced by the processing pipeline.

Nonuniformity corrections are performed with sets of calibration images collected once a month. This includes camera readout bias and dark frame subtraction as well as flat field division. Ideally, some calibration images would be collected on a nightly basis; however, the cameras do not have individual filter wheels that can be changed remotely, so the collection of calibration images requires changing the filter of each camera by hand. Since the cameras are hosted in a remote location, it is not feasible to collect calibration data each night. An analysis of the effect of the age of calibration data on the quality of the results is reserved for future analysis and is beyond the scope of this work.

Image plate solution and photometric calibration are performed using the GAIA DR2 star catalog for reference [28]. The photometric measurements are calibrated using in-frame solar-type stars, chosen based on the criteria published by Andrae et al. [29] and corrected to first order in GAIA G magnitude [27,30,31]. Since each image is quite large (44 deg^2^) and there is the potential to collect tens of thousands of images each night, using in situ network access to the GAIA DR2 catalog is not a feasible option. As such, a custom local star catalog based on GAIA DR2 that is made up of a strip of the sky centered on the GEO belt as seen from Tucson (-5 deg declination) was made to replace the need for catalog hosting and query service such as Vizier. This local catalog contains over 75 million stars and has information for stars as faint as 18 GAIA G magnitude.

Since Stingray’s cameras are mechanically fixed and take 10 s exposures, star trails in the images are approximately 12 pixels long when binned 2×. While not pathologically long, these trails do have the potential to contaminate satellite observations. Special care has been taken to minimize this effect on the results at each step of the processing pipeline, including a final stochastic filtering of the extracted satellite observations, which is designed to reject observations believed to be contaminated by nearby stars in the image. As a result, the total number of observations for each satellite will be reduced in general, but the astrometric and photometric quality of the observations will be more reliable.

Image source centroids are computed using an iterated isophotal first-order moment with the second-order correction given in Equation (1). The iteration is initialized with a simple isophotal first-order moment, ${\;}^{\left(0\right)}{\bar{x}}_{i}$, and follows the scheme

(1) $$\begin{array}{c} {\;}^{\left(k+1\right)}{\bar{x}}_{i}={\;}^{\left(k\right)}{\bar{x}}_{i}+2\frac{\sum_{j}{\;}^{\left(k,j\right)}w^{\left(j\right)}I{(}^{\left(j\right)}x_{i}-{\;}^{\left(k\right)}{\bar{x}}_{i})}{\sum_{j}{\;}^{\left(k,j\right)}w^{\left(j\right)}I} \end{array}$$

where i∈{1,2} denotes the axis of the centroid coordinate (i.e., ‘x’ or ‘y’), *k* is the iteration index, and *j* is the summation index over all pixels that belong to a given image object. The ${\;}^{\left(j\right)}I$ are the pixel values and the ${\;}^{\left(k,j\right)}w$ are given in Equation (2) by

(2) $$\begin{array}{c} {\;}^{\left(k,j\right)}w=\exp\left(-\frac{{\;}^{\left(k,j\right)}r^{2}}{2\sigma^{2}}\right) \end{array}$$

In Equation (2), ${\;}^{\left(k,j\right)}r$ is the Euclidean distance between each pixel in the object and the previous centroid location, and σ is the diameter that contains half of the object flux in the image divided by $\sqrt{8ln2}$. The centroid position is iterated until the change between iterations is less than two ten-thousandths of a pixel (approximately three iterations).

This method is chosen since it is more accurate than a simple isophotal centroid and is faster than using PSF fitting with very similar accuracy (close to the limit of image noise). This process is accomplished with the aid of the open-source software package Source Extractor [32]. Once the pixel locations of the centroid of all light sources in the image are extracted, their relative spacing is cross-correlated with that of stars from the GAIA DR2 star catalog using the open-source software Scamp [33]. This yields the plate solution for a given image describing the mapping between our camera frame on the sky and a topocentric celestial angular coordinate frame (right ascension and declination).

With direct mapping between the camera frame and the celestial coordinates along with very precise centroid information, the next step is to compute the photon flux for each source in the image. Since our observations are taken from a fixed platform, stars are streaked in the images. To account for this, we use an elliptical aperture method to calculate the flux. We estimate the desired aperture to be 2.5 times larger than the FWHM of the average point source PSF in a given frame. The ZWO cameras we use have tunable gain, given in centibels, and, therefore, it is straightforward to calculate the approximate electrons per analog-to-digital units ($e^{-}/\mathrm{ADU}$) and recover the flux from a desired source, as shown in Equation (3).

(3) $$\begin{array}{c} \varphi_{i}=\sum_{\delta }\sqrt{10^{\left(1.39-\frac{G}{100}\right)}}\times c_{\delta j} \end{array}$$

In Equation (3), $\varphi_{i}$ represents the total flux of the ith point source. The summation is over some elliptical region δ where $c_{\delta j}$ are the individual pixel ADU counts within that region. Finally, *G* is the camera gain setting in centibels, which is tunable on the ZWO ASI1600MM Pro cameras used. Using the calculated flux of each point source in a given image, the apparent magnitude of that point source can be calculated by Equation (4). While the precise origin of the stellar magnitude system is somewhat debated, the system ubiquitously in use today in the astronomical sciences (shown in modified form in Equation (4)) was first formalized by Pogson [34].

(4) $$\begin{array}{c} M_{i}=-2.5M_{1}log_{10}\left(\frac{\varphi_{i}}{t}\right)+M_{0} \end{array}$$

**Figure 9:**
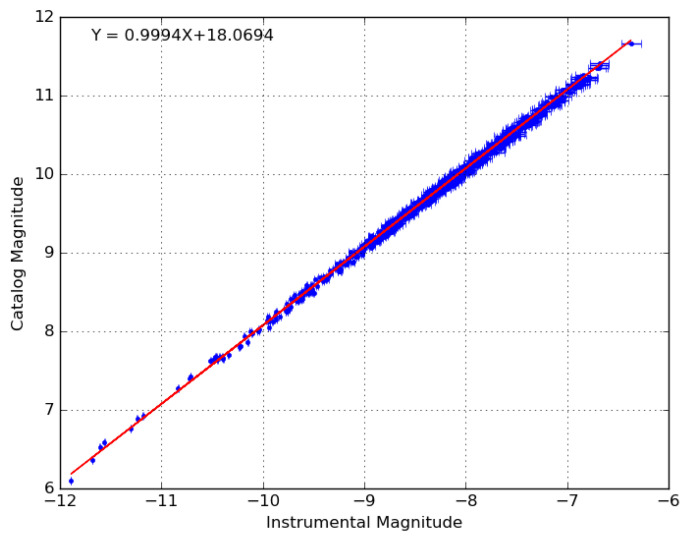
Example of the photometric calibration applied to the observations from each camera. In this example, the RMS error between measured and catalog magnitudes of the stars is 0.04 magnitudes.

Here, $M_{i}$ is the calculated apparent magnitude of the ith point source and $M_{0}$ and $M_{1}$ are the zero-point and first-order corrections that are applied and are calculated separately as follows. The linear photometric correction used to convert from instrumental magnitude to GAIA G magnitude (parameters $M_{0}$ and $M_{1}$) is calculated by using all solar-type stars identified in the set of images after iterative sigma clipping, weighted by the individual flux measurement uncertainties (reciprocal of SNR). Solar-type stars are identified using the GAIA DR2 criteria published in [29], and when multiple measurements of the same star are found across multiple images (depending on the processing mode), a median flux and SNR are used in the fit. The RMS uncertainty quoted is calculated as the root mean squared error between the post-correction measured magnitudes of all solar-type stars in the images (no sigma clipping) and their published GAIA DR2 magnitudes. Figure 9 gives an example of the fit that is performed in order to calculate the photometric correction, where the RMS uncertainty in this case is 0.04 magnitudes.

Once the corrected astrometric and photometric measurements of all sources in an image are made, the next step is to extract the desired information of any RSOs that may be present. Once the data for each object of interest in each of the images for a given night are extracted, the observation data are automatically uploaded to VerSSA (the University of Arizona’s SSA cyberinfrastructure system) for potential further processing (orbit determination, change detection, etc.). The output format of the reduced satellite measurements is flexible, though nominally, the measurements are 80 bytes per observation data point, so typically only a few megabytes of data per night will need to be transferred. This can be adapted to accommodate a real-time or small-batch stream of observations to VerSSA instead of an end-of-the-night accumulation if closer to real-time analysis is desired. The raw image pixel data are stored in local high-density storage systems for up to six months in case there is a need to revisit the analysis at any time.

## 3. Deployment and Performance Evaluation

### 3.1. Commissioning and Calibration

**Figure 10:**
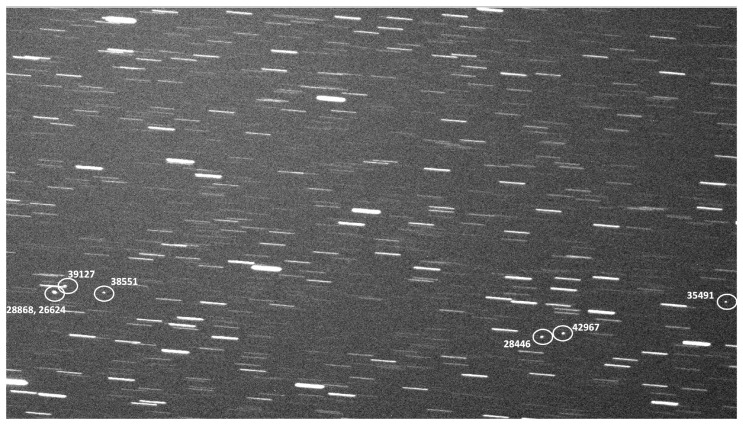
Zoomed in example image from one of the Stingray cameras. Several GEO satellites have been labeled with their NORAD designators. In this frame, two satellites (28868 and 26624) are too close together to be resolved. From left to right, the satellite common names are ANIK F1-R, ANIK F1, ANIK G1, ECHOSTAR 17, AMC-15, ECHOSTAR 105/SES 11, and GOES 14.

Stingray began collecting test data on 16 November 2021, with the system running every night and incorporating any changes to software or hardware as needed in real time. Data were successfully collected for an average of just over 8 h a night for 160 nights until 16 June 2022, when Stingray was temporarily shut down for the local monsoon season. After the monsoon, additional test data were collected until the first night of operation in nominal survey mode on 5 May 2022. In total, 220 nights of test data have been collected with varying operation modes and weather conditions. The initial survey ran for an additional 31 nights. Figure 10 shows a zoomed and cropped example of a Stingray image from a single camera with a 30 s exposure, where some of the GEO satellites in the FOV have been circled and identified.

During the final night of test data collection pre-monsoon (16 June 2022), the Moon was approximately 98% full, and there were some dispersed clouds early in the night. Despite this, the Stingray system was still able to maintain an average photometric calibration error of 0.06 magnitudes across all catalog sources in all images that were successfully plate solved that night. During the first night of operation in the nominal survey mode on 5 May 2023 (one ten-second exposure image 2× binned every minute from each camera), Stingray collected 6476 images. The Moon was again 98% full and was roughly 10 degrees below the GEO belt as seen from Tucson, AZ, throughout the night. Again, the Stingray system was able to maintain an average photometric calibration error of 0.07 magnitudes across all catalog sources in all images that were successfully plate solved. During the 31 nights of the initial survey, the aggregate photometric uncertainty was 0.062 ± 0.008 magnitudes (1σ). Astrometric and photometric measurements were extracted for 142 GEO satellites on the first survey night, averaging 233 observations per satellite.

**Figure 11:**
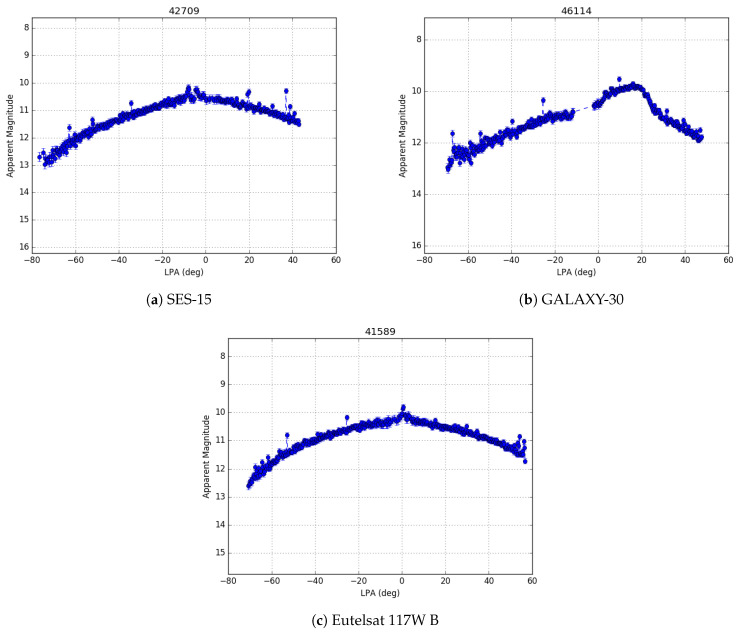
These are example light curves of the three WAAS satellites on 5 May 2023, each seen by a different Stingray camera. SES-15 and Eutelsat 117W B are of the same satellite bus type (BSS-702SP), while GALAXY-30 is a GeoStar-2 bus. The error bars are a sum of the inverse of the SNR of each measurement and the mean RMS residual error in the photometric calibration.

Three GEO satellites (SES-15, GALAXY-30, and Eutelsat 117W B) are part of the Wide Area Augmentation System (WAAS), are used to help improve GPS accuracy, and are within Stingray’s FOR. An example of the all-night photometric measurements of each of these three satellites is given in Figure 11. Being part of the WAAS, each of these satellites has publicly available, high-accuracy ephemeris, which makes them very useful calibration targets. Comparing the ephemeris for SES-15 to the Stingray observations on the final night of the test phase pre-monsoon on 16 June 2022, the average error in declination measurement was found to be consistent with zero (eight-thousandths of a pixel) with a standard deviation of approximately 0.1 pixels, which is consistent with sub-pixel centroiding limits. The average error in right ascension, however, was found to be biased by approximately two pixels (∼24 arc-sec) with a standard deviation of approximately half of a pixel. Given that SES-15 has a near-zero inclination (<0.02 deg), the right ascension error can be considered analogous to a time error, which, in this instance, corresponds to a timing error of 1.6 s. Performing a similar analysis on GALAXY-30, seen from a different Stingray camera controlled by the same computer, shows a similar timing error of 1.2 s.

The time on each of the Stingray computers is controlled by a background process that synchronizes with a NIST time server every minute, while network latency and system clock drift may play a part in this error (likely at most a few tenths of a second), it is unlikely this is the sole cause of the error. There will also be some latency in the image capture, readout, and file creation process that is not reflected in the time information in the image meta data. As such, a new keyword was added to the meta data in each image header that not only gives the requested start time of the exposure but also the readout time of the image frame. If there is no latency in the process, then the difference between these two times will just be the desired exposure time. After the addition of this keyword, however, it is apparent that this is not the case. There is typically some random additional latency on top of the desired exposure time. Using average corrections from this additional time information in the image meta data, the error analysis shows greatly improved results.

Comparing the ephemeris of all three WAAS satellites to the measurements taken by Stingray on the first nominal survey night (5 May 2023) shows that there is no longer an appreciable bias in the right ascension error. In fact, both the right ascension and declination errors are approximately statistically consistent with zero and at the theoretical limits of sub-pixel centroid calculation. Table 3 gives a summary of the error analysis results for the three WAAS satellites on 5 May 2022. An average exposure latency correction is used for each satellite instead of a correction per measurement as there is still additional work to do in characterizing more precisely how this latency affects the measurements. An average correction reflects a best effort in this case while more analysis is conducted. In the future, it is expected that the precision of the measurements will be increased once this system artifact is further characterized.

In order to calculate the results in Table 3, the published WAAS ephemeris has to first be converted into pseudo-observations corresponding to the epochs of each measurement. After parsing the WAAS message format, the ephemeris is given as state vectors in an Earth-centered, Earth-fixed (ECEF) reference frame at some time interval. First, a cubic spline is fitted to the time history of each component of the state vectors separately. This spline is then sampled at each of the observation epochs. The observer position is also calculated in the same ECEF reference frame using an oblate spheroid model for the Earth. Both the satellite state vectors, and the site location vector are then rotated to the mean equator mean equinox (MEME) J2000 reference frame consistent with the IAU-76/FK5 reduction (same frame as the observations). Light has an appreciable travel time when considering GEO satellites (∼0.12 s), so this must be accounted for in both the observer and satellite positions at the epoch. The exact light travel time is typically calculated by iteration; however, here it is calculated in a single pass, since the changes after the initial calculation are very small when compared to other sources of error, and the calculation typically converges in 2–3 iterations anyway. Once the light travel time has been taken into account, the satellite ephemeris positions must be corrected to apparent positions by accounting for annual stellar aberration. This is another non-Newtonian effect that must be accounted for and is due to the fact that the Earth is not a true inertial frame of reference and is, in fact, moving quite quickly. After these corrections, topocentric MEME J2000 right ascension and declination may be calculated at each observation epoch and compared to the Stingray measurements. The ephemeris of the WAAS satellites is published in one-day intervals and is typically made available 24 h later. As such, this analysis is a useful process to periodically check the astrometric accuracy of the Stingray results to ensure consistency.

### 3.2. Sample Light Curves

**Figure 12:**
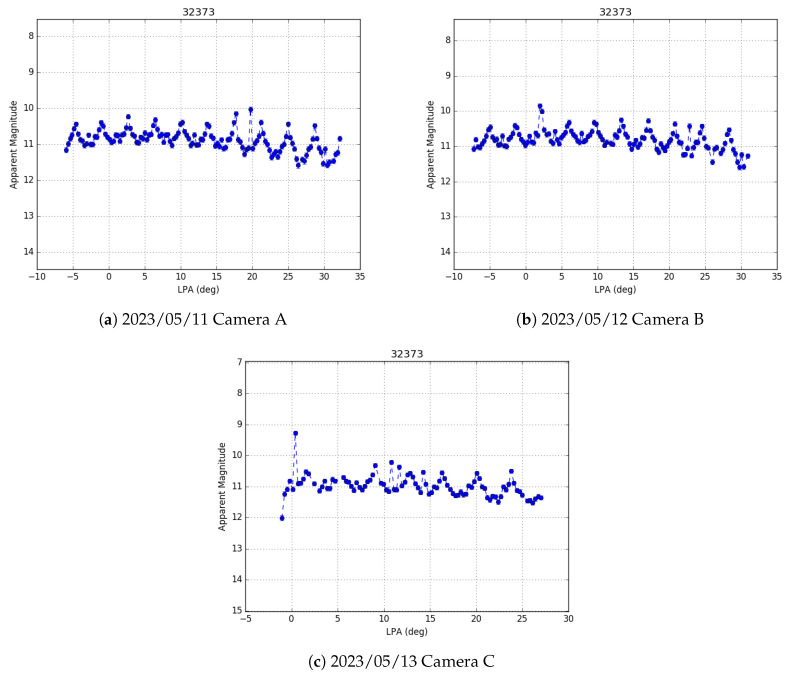
Light curves of Raduga 1M-1 (NORAD ID 32373), a defunct Russian military communication satellite now tumbling, as can be seen by the periodic light curves. This debris satellite was drifting through the GEO belt and was seen by three different Stingray cameras on three consecutive nights as it drifted westward in the sky. The error bars are a sum of the inverse of the SNR of each measurement and the mean RMS residual error in the photometric calibration.

Raduga 1M-1 (NORAD ID 32373) is a Russian military communication satellite that was launched on 9 December 2007 into geostationary Earth orbit. Its mission lifetime was ten years, after which it was moved into a graveyard orbit approximately 170 km beyond the GEO belt, giving it an orbital period of just over 24 h. As a result, Raduga 1M-1 is currently inclined by almost 8 degrees and drifts westward in the sky at a rate of approximately 3 degrees per day. Since the satellite is no longer in operation, its orientation is no longer controlled, and it is free to rotate and tumble due to perturbations in its environment. Shown in Figure 12 are three examples of light curves collected of the Raduga 1M-1 satellite as it drifted across the Tucson sky. These light curves were collected on three consecutive nights (11–13 May 2023) as the satellite moved between the FOV of three different cameras.

**Figure 13:**
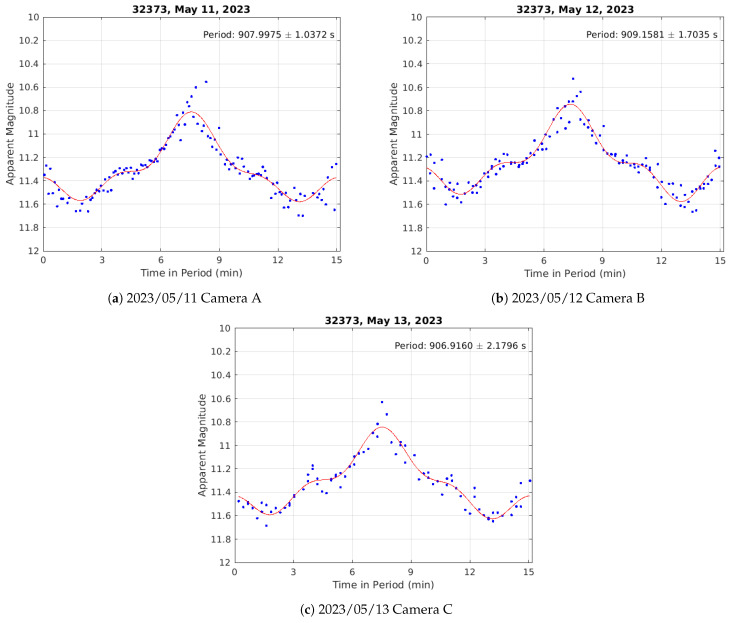
Light curves of Raduga 1M-1 (NORAD ID 32373) on three separate nights after performing a four-parameter Fourier period fitting analysis. The periods in each case are given in seconds with their associated 1σ bounds.

In contrast to the light curves of the three WAAS satellites shown in Section 3.1 (Figure 11), which are of three-axis stabilized GEO spacecraft, the light curves of Raduga 1M-1 are clearly quasi-periodic on a short (order of minutes) time scale. This periodicity implies that the spacecraft is no longer stabilized but instead is rotating (or tumbling) uncontrolled. Performing a four parameter Fourier fit, as shown by Campbell et al. [22], we can find the period of rotation for all three nights of data. Shown in Figure 13 are the results of the Fourier period analysis. The calculated periods of the light curves for each night, along with their associated 1σ uncertainties are also given in the figure. The conflated rotation period of the estimated distributions is 908.1135 ± 0.8207 s.

There are a variety of other analysis techniques that could be applied to these data to try and learn more about the Raduga 1M-1 satellite [4,17,18,19,21], including many not mentioned here. Further analysis is out of the scope of this work; however, this gives us a glimpse into the wealth of potential research that the Stingray system can provide. This example is of just a single satellite among the hundreds of RSOs observed by Stingray every night.

## 4. Summary

The novel Stingray sensor system is designed to collect data en masse on artificial satellites in the near-geosynchronous Earth orbit every clear night visible from Tucson, Arizona. Stingray is comprised of 15 ASI 1600 MM Pro cameras that are mated to Sigma 135 mm f/1.8 lenses and are controlled simultaneously by four separate computers. Each camera is fixed in position and images a 7.6 by 5.8 degree portion of the GEO belt for a total of a 114-by-5.8-degree field of regard. The system is designed to be completely automated in both data acquisition and processing, with human oversight reserved for data product quality assurance and system maintenance. The photometric and astrometric measurements of all near-GEO satellites visible to the Stingray system each night (∼200) can be available in near-real time, the access to which has never before been available to researchers and opens the doors for many new types of RSO characterization and analysis.

The immense volume of data produced by Stingray is ideal for training machine learning models, which historically have been primarily trained on simulated data due to the volume of data required and the comparatively low throughput of traditional telescope systems. Implementing commercial off-the-shelf hardware components in such a unique way has necessitated the bespoke handling of nearly all aspects of control and data processing to ensure both the reliable autonomy and functionality of the system. After over 250 nights of testing, the results from the initial survey exhibit absolute accuracy in astrometric measurements approaching theoretical limits and photometric calibration to approximately six hundredths of an order of magnitude. These extremely impressive results for a bespoke automated system designed to collect data en masse will ensure high-quality and reliable data for a plethora of future RSO characterization research topics.

## Figures and Tables

**Table 1:** Details of the lenses considered during the Stingray hardware trade study.

Manufacturer	Focal Length (mm)	Aperture (mm)	f/#	FOV (${\;}^{\circ }$)	Pixel Size ($\frac{\mathrm{as}}{\mathrm{px}}$)	Limiting Mag	Units Req
Canon	85	47	1.8	12 × 9	9.3	12.5	10
Canon	85	60	1.4	12 × 9	9.3	13.2	10
Canon	135	67	2	7.6 × 5.8	5.9	13.9	16
Canon	200	71	2.8	5.1 × 3.9	8.5	14.5	24
Canon	200	100	2	5.1 × 3.9	8.5	14.9	24
Rokinon	85	60	1.4	12 × 9	9.3	12.75	10
Sigma	105	75	1.4	9.7 × 7.4	7.6	13.5	12
Sigma	135	75	1.8	7.6 × 5.8	5.9	14.5	16

**Table 2:** Relevant physical parameters for the Stingray system.

Name	Camera	Focal Length (mm)	Aperture (mm)	FOV (${\;}^{\circ }$)	FOR (${\;}^{\circ }$)	Pixel Scale ($\frac{\mathrm{as}}{\mathrm{px}}$)
Stingray	15× ZWO ASI1600MM Pro	135	75	7.6 × 5.8	114 × 5.8	5.9

**Table 3:** Astrometric error analysis results comparing high-accuracy ephemeris of WAAS satellites to Stingray measurements.

Satellite	Error in Right Ascension (arc-sec (px))	Error in Declination (arc-sec (px))
SES-15	0.653±1.635 (0.055±0.139)	−0.300±1.756 (−0.025±0.149)
GALAXY-30	0.917±2.034 (0.078±0.172)	−0.071±1.815 (−0.006±0.154)
Eutelsat 117W B	−0.279±1.461 (−0.024±0.124)	1.614±1.563 (0.137±0.132)

## Data Availability

Data available on request.
